# Attitudes toward using “Simple simulator for calculating nutritional food stocks in preparation for large-scale disasters” among local governmental personnel and public health dietitians in Japan: An explanatory mixed methods study

**DOI:** 10.3934/publichealth.2022051

**Published:** 2022-11-11

**Authors:** Noriko Sudo, Nobuyo Tsuboyama-Kasaoka, Ikuko Shimada, Keiichi Sato, Akiko Kubo

**Affiliations:** 1 Natural Science Division, Faculty of Core Research, Ochanomizu University, Bunkyo City, Tokyo 112–8610, Japan; 2 Section of Global Disaster Nutrition, International Center for Nutrition and Information, National Institute of Health and Nutrition, National Institutes of Biomedical Innovation, Health and Nutrition, Shinjuku City, Tokyo 162–8636, Japan; 3 Department of Nutrition, Faculty of Nutrition, University of Kochi, Kochi City, Kochi 780–0515, Japan; 4 School of Network and Information, Senshu University, Kawasaki City, Kanagawa 2148580, Japan; 5 Kagawa Nutrition University, Sakado City, Saitama 350–0214, Japan

**Keywords:** food stockpile, nutrition assistance, Japan, natural disaster, public health dietitian

## Abstract

The Japanese Ministry of Health, Labour and Welfare compiled an Excel sheet — “Simple simulator for calculating nutritional food stocks in preparation for large-scale disasters” (Simulator). We examined the level of recognition and use of the Simulator by local governments and identified the points for its improvement. In stage 1, we surveyed local government personnel who participated in the “Workshop for nutrition assistance during large-scale disasters” held in November 2020 (n = 458; 313 responded) with an online questionnaire on the use of the Simulator and associated issues. Stage 2 involved group interviews with 15 public health dietitians who had been involved in food assistance during past natural disasters to identify points for improving the Simulator and the problems with food assistance during natural disasters. In stage 1, 233 responders (74.4%) confirmed their awareness of the existence of the Simulator. While 85 individuals (36.6%) used the Simulator, 63 individuals (74.1%) confirmed that they would use it in the future to plan and evaluate local government stocks. In stage 2, multiple comments regarding the Simulator's applicability and improvement in a realistic situation were collected. In order for the administrative staff in charge of disaster management to understand the nutritional importance of stockpiling main/side dishes, it was suggested that specific combinations of foods that meet the required amounts should be shown and that visualization using food products and the number of people covered by stockpiled foods may be easier to understand than pure nutritional values.

## 1. Introduction

At the Tokyo Nutrition for Growth Summit that was held in December 2021, the Government of Japan introduced other countries to Japan's “Nutrition Policy to Leave No One Behind” [1]. Japan's appeal lies not only in its world-leading universal healthcare insurance system, but also in the fact that in addition to medical insurance for those who become ill, it provides primary prevention (health promotion) and secondary prevention (early detection and treatment of diseases) for all life stages from infancy to old age. These services are provided free of charge at the municipal level, which is closest to citizens, and they include health and nutrition counseling as well as medical checkups by dietitians and other specialists stationed at municipal community health centers. Simultaneously, a nutrition policy for the injured, sick, or those affected by a disaster is being created. In addition to the deployment of registered dietitian nutritionists at medical institutions under the Health Promotion Act, there are initiatives for the provision of healthy food at shelters even during large-scale disasters.

In the Great East Japan Earthquake, however, water and food were lacking at shelters [2]. The Fukushima Health Management Survey also showed that people living in evacuation shelters or temporary housing had poor dietary intake of fruits and vegetables (non-juice), meat, soybean products, and dairy products [3]. The need for improvement of meals provided at evacuation shelters should be addressed not only by Japan but also globally. Poor food was reported by Hurricane Katrina evacuees in Houston and New Orleans shelters [4],[5]. Pregnant and postpartum women affected by Typhoon Haiyan, which hit the Philippines, suffered from hunger and food shortage and had to look for food for survival [6]. Considering that many evacuees have a difficulty to find food, their vital needs should be supplied efficiently throughout the disaster and post-disaster periods by local authorities. Without an adequate stock of foods, satisfying the daily nutritional requirement of the victims without disruption might be problematic [7].

According to the World City Risk 2025 [8], Tokyo has the highest risk of flooding and tsunamis and the sixth highest risk of earthquakes among the world's major cities. The probability of an earthquake directly hitting the Tokyo metropolitan area within the next 30 years could be as high as 70% [9]. Japan is particularly prone to disasters and its cities, towns, and villages establish evacuation shelters when such a disaster strikes. At these shelters, evacuees are given food stockpiled by the municipalities until the arrival of relief supplies. If these stockpiles are insufficient, prefectural stockpiles are released, and if that is insufficient, an appeal is made for relief supplies from the national government. In a compound disaster covering a wide area, such as the Great East Japan Earthquake, it took five days for relief supplies to reach the disaster zone: three days to procure food and two days to secure transportation routes and vehicles [10]. In this context, the difficulty in providing food supplies to a disaster-stricken area during five days from the disaster emphasizes the importance of using food stockpiles prepared in advance. Disasters and extreme climate events in recent years help many countries to justify emergency food reserves [11]. In a global view point, food assistance aims to ensure the consumption of sufficient, safe, and nutritious food in anticipation of, during, and in the aftermath of a humanitarian crisis when food consumption would be otherwise insufficient or inadequate to avert excess mortality and emergency rate of acute malnutrition [12]. However, current provision of emergency food assistance remains unclear in terms of targeting energy and nutrient content of the food rations. The United States Agency for International Development published a report aiming to improve nutritional quality of food in assistance program [13]. A high energy ratio of lipids and carbohydrates along with insufficient amounts of vitamins and minerals in food stockpiles of local governments in Japan was reported [14]. Some researchers evaluated or designed food assistance based on nutritional guidelines [15] and food guide [16].

As a practical tool to showcase Japanese natural disaster countermeasures, the Ministry of Health, Labour and Welfare (MHLW) compiled an Excel sheet — “Simple Simulator for Calculating Nutritional Food Stockpiles in Preparation for Large-scale Disasters” (Simulator) — to be unveiled at the Tokyo Nutrition for Growth Summit. In April 2020, local governments were notified about this Simulator ahead of the Nutrition for Growth Summit, so that it could be used for stockpiling plans under normal conditions [17]. This is the world's first attempt by the national government to develop an assistance tool for improving local governments' food stocks. However, the levels of recognition and use of the Simulator by local governments have not been evaluated so far. To preserve life by minimizing preventable mortality in early emergencies, nutritional consideration to food stocks is essential. Japan's experiences in the development and improvement of the Simulator based on the feedback from local government officials might provide useful information for other countries that face similar challenges. In addition to reporting the level of recognition and use of the Simulator by local governments, this study used a questionnaire and group interviews to identify points for improving the Simulator and problems with food assistance during natural disasters. The end goal of this study was to reveal how the Simulator should be revised for better use. Since humanitarian response, particularly for food and nutrition, must be based on best practices, this study could contribute to a similar project in other countries. This study would be of importance to stakeholders involved in disaster management, especially for improving the quality and quantity of local food supplies that can be used before any external help can reach the disaster-affected area.

## 2. Materials and methods

This was an explanatory mixed methods study. In stage 1, we surveyed local government personnel who participated in the “Workshop for nutrition assistance during large-scale disasters” held by the Japan Public Health Association in November 2020 (n = 458; 313 responded) with an online questionnaire on the use of the Simulator and associated issues. Stage 2 involved group interviews with 15 public health dietitians who had been involved in food assistance during past natural disasters to identify points for improving the Simulator and the problems with food assistance during natural disasters. Since this was a mixed study consisted of a preliminary questionnaire survey and qualitative interviews, no statistical analysis and power calculations were applicable.

### 2.1. Japanese government organization

**Figure 1.**
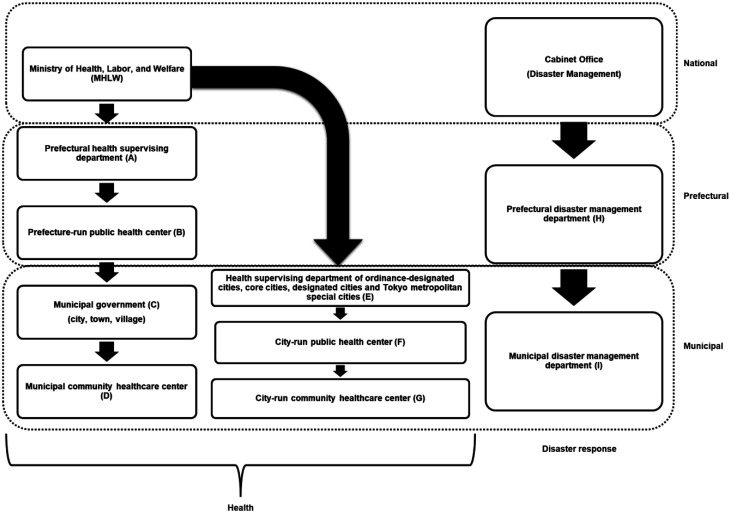
Flow of information from national government in Japanese administrative organizations.

Disaster prevention and response to natural disasters in Japan involve many ministries and governmental agencies. At the national level, the earthquake-proofing and upkeep of roads and bridges are under the jurisdiction of the Ministry of Land, Infrastructure, Transport and Tourism; food stockpiles and food support are under the jurisdiction of the Ministry of Agriculture, Forestry and Fisheries; medical care in natural disasters and the health and welfare of affected people fall under the jurisdiction of the MHLW; and aid during natural disasters and the delivery of relief supplies using the Japan Self-Defense Forces are under the jurisdiction of the Ministry of Defense. The Cabinet Office's Disaster Management Team is in charge for coordinating these responses among different ministries and agencies. At the local government level, the natural disaster-response offices of the prefectural and municipal governments serve as coordinators. Food stockpiles and procurement are taken care of by the disaster-prevention departments, whereas public health activities for maintaining the health of affected people are carried out by health departments. Notices and information from the national government to each of these offices are sent through separate channels (Figure 1).

Cooperation that traverses departments is difficult with the vertical division from nation to municipality.

### 2.2. Simple Simulator for calculating nutritional food stocks in preparation for large-scale disasters

**Figure 2.**
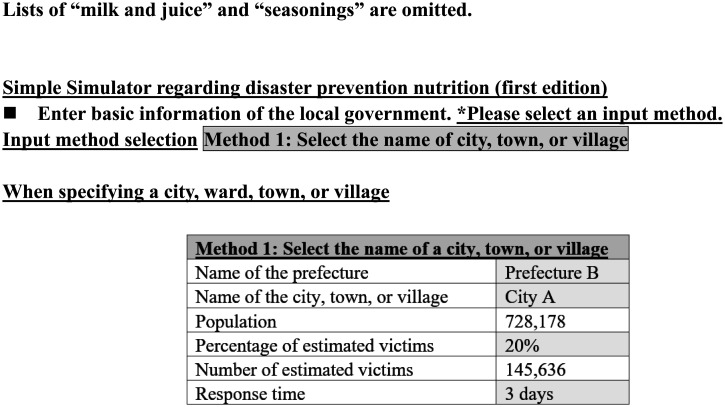
Window for selecting municipality's name, estimated percentage of victims, and number of days for stockpiles. (This corresponds to the steps 1–3 in Table 1).

**Figure 3.**
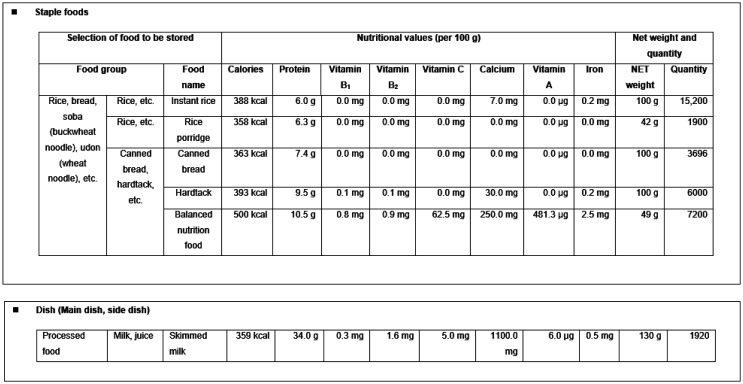
Stockpile food selection window. (This corresponds to the steps 4 and 5 in Table 1).

**Figure 4.**
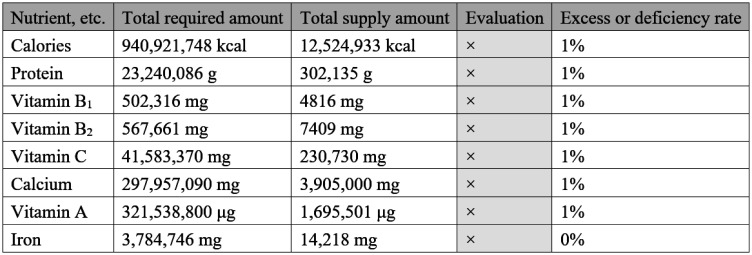
Amount of nutrients necessary for estimated victims and amount of nutrients supplied by the stockpiles. (Total required amount: Dietary reference intake value (per day), weighed by the gender and age group composition of the municipality in question, multiplied by the number of days covered and number of victims; Total supply amount: Amount of nutrients supplied by the municipality in question's food stockpile; Excess/deficiency ratio (%) = Total supply ÷ Total required × 100; Evaluation: If the excess/deficiency ratio is more than 100%, Ο will be displayed, and if it is less than 100%, × will be displayed).

The outline of the Simulator produced by the MHLW is shown in Tables 1–3 and Figures 2–4. The notice publicizing the URL from which the Simulator can be downloaded was sent to the health and welfare offices (A and E in Figure 1) of local governments in April 2020.

**Table 1.** Outline of the Simulator for calculating nutritional food stockpiles in the preparation for large-scale disasters.

Number	Content
1	Population is displayed when the name of a municipality is selected (in Figure 2, “City A, Prefecture B” is selected).
2	The number of people to be covered by the public stockpiles is displayed when the estimated percentage of affected people is entered (default value of 20%).
3	Enter the number of days for which the stockpile is to be used (default value of 3 days).
4	Select stockpile foods from the food-product list using a pull-down menu. The food-product list is divided into “staple foods” (Table 2) and “main and side dishes” (Table 3). “Method of use” explains that “nutrients contained in the main and side dishes, such as vitamins B_1_ and B_2_, are important for burning carbohydrates, so food products are to be selected with consideration for the type of staple food/main and side dishes.”
5	Input the quantity of each food product to display the nutrient content used in the Standard Tables of Food Composition in Japan (Figure 3).
6	Amounts of calories and nutrients, calculated by weighing the values of the Dietary Reference Intakes (per day) by the gender and age group composition of the city/town/village, multiplied by the number of days covered and the number of affected people is shown in Figure 4. Additionally, excess/deficiency ratio (%) is given.
7	A ratio of 100% or above is marked with “Ο,” whereas a ratio of less than 100% is marked with “×.”

**Table 2.** List of food items for “staple food.”

On the list of food items, you may choose from the four groups that fall under the category of “staple food”: “rice and the like,” “wheat flour, rice flour, rice cakes,” “dried noodles, instant noodles, cup noodles,” and “dried bread, cookies, and the like.” As these items are essential for energy intake in a disaster, please ensure that you have sufficient stock. It is also critical to stockpile not only dry bread, cookies, and cup noodles, but also rice and the like, considering the needs of individuals with wheat allergies.

**Table:** <Staple foods>

Rice and the like (10 items)	Wheat flour, rice flour, rice cakes (10 items)	Dried noodles, instant noodles, cup noodles (13 items)	Dried bread, cookies, and the like (6 items)
Alpha rice	Wheat flour	Instant Chinese noodles	Hardtack
Porridge	Premix flour for okonomiyaki	Chinese-style instant cup noodles	Canned bread
Brown rice	Premix pancake flour	Chinese-style instant cup Yaki-soba noodles	Cookies
Half-polished rice	Wheat germ	Japanese-style instant cup noodles	Cereal
70% polished rice	Rice cakes	Barley, dried barley noodles	Sweet red bean jelly (Youkan)
Polished, white, ordinary rice	Top-grade rice flour made from non-glutinous rice	Dried udon noodles	Balanced, nutritional food
Polished, white, glutinous rice	Brown rice flour	Dried Somen/Hiyamugi noodles	
Polished Indica rice	Rice flour	Dried Chinese noodles	
Polished whole rice with germ	Glutinous rice powder	Dried Okinawa soba noodles	
Germinated brown rice	Rice bran	Dried macaroni, spaghetti	
		Soba noodles, dried soba noodles	
		Rice flour	
		Rice noodles	

**Table 3.** List of processed food items for “main and side dishes.”

On the list of food items, you may choose from the five groups that fall under the categories of “Main and Side Dishes”: “pouched, sterilized food,” “canned food,” “dried food,” “milk and juice,” and “seasonings.” Meals based on staple foods lack nutritional balance, so it is important to ensure nutrition by also having canned foods and other “side dishes” in stock.

**Table:** <Processed foods items for main and side dishes>

Pouched, sterilized food (9 items)	Canned food (29 items)		Dried food (47 items)	
Pouched beef curry	Canned sardines	Canned corned beef	Dried gourds	Dried whole Urume sardines
Pouched beef stew	Canned tuna	Canned grilled chicken	Dried taro stems	Small, boiled–dried Katakuchi sardines
Corn cream soup powder	Canned salmon, trout	Canned quail eggs in water	Dried osmund	Dried whole Japanese pilchard
Pouched corn cream soup	Canned mackerel	Canned whole eggs in water	Dried daikon strips	Dried bonito flakes
Curry roux	Canned Pacific saury	Canned boiled yellow soybeans	Dried Chinese chili peppers	Dried flatfish
Hashed beef roux	Canned tuna in water	Canned boiled adzuki beans	Dried bracken	Dried, seasoned silver-stripe round herring
Salmon flake and green tea rice seasoning mix	Canned tuna in oil	Canned boiled asparagus in water	Dried wood ear mushroom	(sardines and the like) Dried blue mackerel flakes
Instant clear soup	Canned clams	Canned boiled green peas in water	Dried Shitake mushrooms	(sardines and the like) Sundried sliced mackerel
Rice dried topping powder	Canned boiled abalone in water	Canned bamboo shoots in water	Dried Maitake mushrooms	Sundried, sliced Pacific saury
	Canned smoked oyster in oil	Canned sweet corn	Natural-dried sea lettuce	Sundried, sliced, Mirin-seasoned Pacific saury
	Canned bamboo shoots (seasoned)	Canned whole tomatoes	Dried steamed Arame seaweed	Dried Pacific cod
	Canned scallops in water	Canned boiled Nameko mushrooms in water	Natural-dried Nori	Sundried, sliced-open herrings
	Canned boiled snow crab in water	Canned boiled mushroom in water	Natural-dried kelp	Dried herring roes
	Canned boiled king crab in water	Canned bee larva	Flaked kelp	Dried abalone
	Canned seasoned squid and the like		Natural-dried red algae	Boiled–dried scallop adductors
			Stainless-kettle dried starry elm	Sakura shrimps
			Iron-kettle dried starry elm	Dried shrimps
			Natural-dried Monostroma nitidum	Dried squid
			Natural-dried Funori seaweed	Shredded dried squid
			Natural-dried Coontail	(squid and the like) Smoked squid
			Natural-dried Wakame seaweed	Dried whole eggs
			Natural-dried Wakame seaweed in water	Dried egg yolk
			Dried Wakame stems	Dried egg white
			Dried boiled infant Pacific sand eel	

### 2.3. Stage 1: Questionnaire survey

#### 2.3.1. Survey period and method

An anonymous online survey was conducted among local government personnel who participated in the “Workshop for nutrition assistance during large-scale disasters” held by the Japan Public Health Association via Zoom on November 9, 2020. Of 458 participants, 313 returned the survey. They were informed about the post-workshop survey and its purpose when they applied to the workshop, and participation in the survey was voluntary. The primary purpose of the survey was to collect feedback from the participants for evaluation of the workshop. There were questions on the use of the Simulator and any associated issues since the workshop included a lecture by the MHLW's nutrition officer about the Simulator.

#### 2.3.2. Questionnaire topics

The first, second, and fifth authors designed the related part of the questionnaire. In addition to the attributes of the respondent, such as their affiliated department, area of responsibility, and position, the questions shown in Table 4 were asked regarding the Simulator.

**Table 4.** Questions about the Simulator.

Number	Content
Q1	Did you know about the existence of the Simulator? (Yes/No)
Q2	(Only for those who said “Yes” to Q1) Have you ever used (entered information into) the Simulator? (Yes/No)
Q3	(Only for those who said “Yes” to Q2) Do you think you will use the Simulator in future to plan and evaluate local government stockpiles? (Yes/No)
Q4	(Only for those who said “No” to Q3) Multiple choices for the reason why you will not use it in the future (Options shown in Figure 5)
Q5	Multiple choices for ideas for improving the Simulator (Options shown in Figure 6)
Q6	(Only for those who said “No” to Q2) Multiple choices for the reason why you have not used it (Options shown in Figure 7)

**Figure 5.**
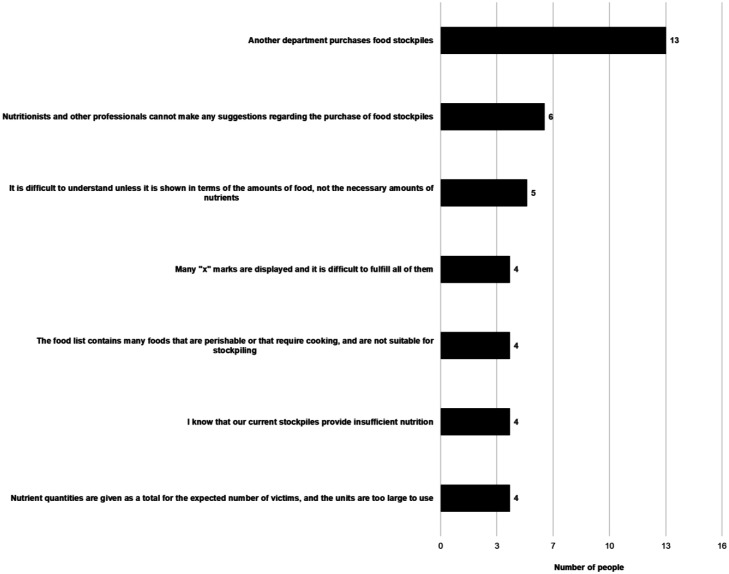
Reasons why respondents do not think they will use the Simulator in the future. (Q4, N = 22, multiple answers allowed)

**Figure 6.**
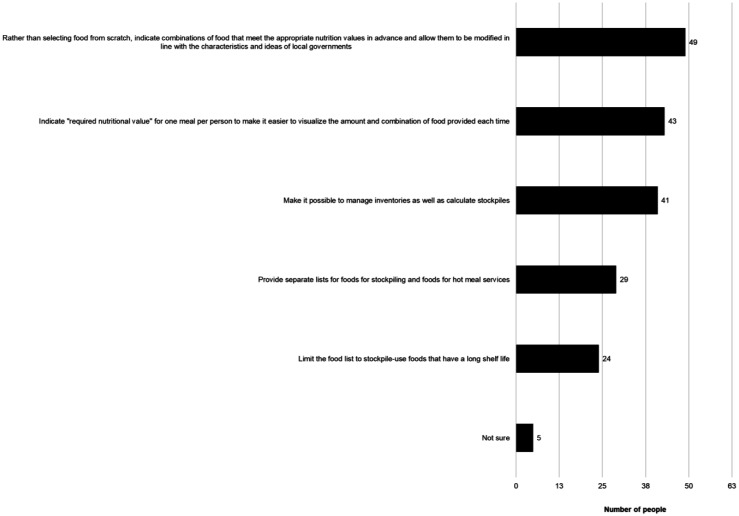
Good ideas for improving the Simulator. (Q5, N = 85, multiple answers allowed)

**Figure 7.**
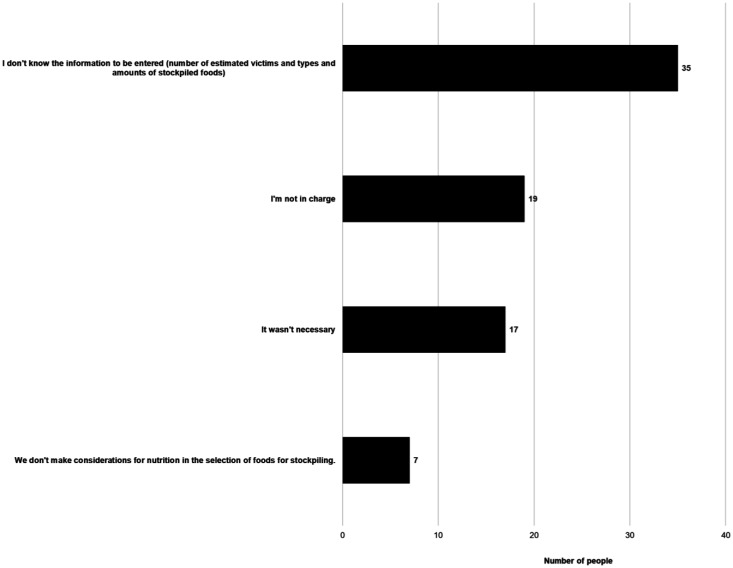
Reasons why the respondents did not use the Simulator. (Q6, N = 147, multiple answers allowed)

### 2.4. Stage 2: Group interviews

#### 2.4.1. Participants and implementation dates

We selected three prefectures, each of which was affected by one of the three major disasters in the 2010s. There were torrential rains in Prefecture X. In Prefecture Y, an earthquake occurred concomitant with a tsunami buffeting the coast, and Prefecture Z experienced an earthquake. Although each disaster damaged several prefectures, Prefecture X was selected because it had the largest number of cities with flood. Prefecture Y was chosen because it had the largest number of deaths. Prefecture Z was selected due to the largest damage by the earthquake.

From September 2020 to December 2020, we conducted group interviews with 15 public health dietitians in each of our chosen prefectures. We selected these dietitians by asking three dietitians currently working in the relevant prefectural governments. The prefectural dietitian in Prefecture X was an acquaintance of the first author, one in Prefecture Y was referred by a nutrition officer of MHLW, and one in Prefecture Z was an acquaintance of the fifth author. They were asked to select those who, at the time of the disaster, had provided support to disaster victims as public health dietitians in each organization (Table 5), and who were thought to be able to give useful interviews. All the dietitians approached by the three prefectural dietitians accepted to participate in the interview.

**Table 5.** Number and categorization of local government dietitians who participated in group interviews.

	Heavy rain (Prefecture X)	Earthquake/tsunami (Prefecture Y)	Earthquake (Prefecture Z)	Total
Prefectural Government	2	2	1	4
Prefecture-Run Public Health Center	1	1	1	3
City-Run Public Health Center	1	0	1	2
City Government	1	1	1	3
Town Government	0	1	1	2
Total	5	5	5	15

The interview for Prefecture X was conducted via Zoom on September 30, 2020. The interview for Prefecture Y was conducted via Zoom on October 1, 2020. The interview for Prefecture Z was divided into two sessions to comply with the participants' availability. The first one was conducted via Zoom on November 19, 2020, with a total of three participants: one from the prefectural government, one from a prefecture-run public health center, and one from a city-run public health center. The second interview with one city and one town dietitian who were unable to attend the first interview was conducted via Zoom on December 4, 2020. All authors except the fifth one were present for each interview. The first author, an associate professor with a PhD and qualification of registered dietitian nutritionist at the time of the study, took the lead in the interviews based on an interview guide. She served as the interviewer since she had experiences of 10 interviews before this [18]–[23].

#### 2.4.2. Interview details

Each group interview was approximately two hours long, but only the part relating to the Simulator was reported in this article.

The first question on the Simulator was “Did you know about the Simulator?” followed by “If so, have you ever used it?” Those who had used it before were asked how easy it was to use it and identify any areas for improvement. Those who had never used it were asked about the areas of improvement after seeing the demonstration of the Simulator by the first author.

The interviews were recorded using Zoom's recording function and transcribed by specialists. The transcriptions were checked by the participants, and their statements were extracted and grouped according to similarity of content. Subheadings were applied to the results reflecting the content of the group, and the speaker and statement were shown in an itemized form.

#### 2.4.3. Interpretive description approach

The main purpose of this mixed methods design case study was to collect ideas how the Simulator can be improved so that it can be more practical and useful in food assistance during natural disasters. We did not employ coding of the transcriptions since coding of vast quantities of overly small data units such as words or phrases could not produce sound and usable knowledge for this purpose. In order to capitalize the earthbound concrete realities of the practice context, we used interpretive description approach [24]. This approach involves researchers who work closely with practitioners in order to examine their practices, with the intention of improving them [25]. In this study, we documented the ideas associated with this phenomenon, thereby revealing the details of the practitioners' involvement. Researchers must play an active role in determining the data that are meaningful, while also reflecting constantly on the nature of that meaning. Accordingly, the first step in data analysis was to look at the data and see what captures the researchers' attention. By collating the data into a number of groups, we were able to obtain a clear meaning of the relationships that exist among them.

#### 2.4.4. Ethics approval of research

This study was reviewed and approved in accordance with the provisions of the Ethics Committee for the Studies of Humanities and Sciences at Ochanomizu University (Notification No. 2020-35) and the institutional ethics committees of the National Institutes of Biomedical Innovation, Health and Nutrition (Notification No. KENEI-139). In stage 1, responses to the post workshop questionnaire were voluntary and no personal information, including the names of the respondents, was collected. In stage 2, a letter of request, an interview guide, and a research cooperation consent form were mailed to the group interview participants in advance, addressed to the individuals and heads of their affiliated organizations. Signed consent was obtained from the participants before the interviews. To avoid the identification of the local governments in question, the date and name of the disasters were not given.

## 3. Results

### 3.1. Questionnaire

#### 3.1.1. Attributes of respondents

Of the 458 workshop participants, 313 responses were received (response rate: 68.3%). The most common affiliation of the respondents was prefecture-run public health center (Figure 1B) with 149 individuals (47.6%), followed by municipal community healthcare center (Figure 1D) with 65 individuals (20.8%). Healthcare was the main area of responsibility of the respondents (287 individuals, 91.7%), followed by disaster prevention (5 individuals, 1.6%). Considering that the topic of the workshop focused on nutrition assistance during natural disasters, most participants were public health dietitians (298 individuals, 95.2%), whereas public health nurses and administrative workers were less represented (8 and 7 individuals, respectively).

#### 3.1.2. Awareness and use of the Simulator

A total of 233 responders (74.4%) indicated that they were aware of the existence of the Simulator. Of these, 232 responded on the use, among which 85 individuals (36.6%) affirmed to have used it, i.e., entered information into the Simulator.

#### 3.1.3. User impressions and ideas for improvement

Of the 85 people who had used the Simulator, 63 individuals (74.1%) confirmed that they would use it in the future to plan and evaluate local government stocks. Figure 5 shows the reasons selected by the remaining 22 individuals who responded that they would not use the Simulator in the future. The reason chosen by the largest number of the respondents was “Another department purchases food stockpiles.” The second most common reason for not intending to use the Simulator in the future, despite having used it before, was formulated as “Nutritionists and other professionals cannot make suggestions regarding the purchase of food stockpiles.” Other reasons included “the food list contains many foods that are perishable or that require cooking and are not suitable for stockpiling.”

Figure 6 shows the ideas for further improvements of the Simulator as provided by 85 individuals who had used the Simulator. The most selected idea was “Rather than selecting food from scratch, indicate combinations of foods that meet the appropriate nutrition values in advance and allow them to be modified in line with the characteristics and ideas of local governments,” followed by “Indicate ‘required nutritional value’ for one meal per person to make it easier to visualize the amount and combination of food provided each time.” Answers regarding the food list were also chosen as ideas for improving the Simulator, such as “Provide separate lists for foods for stockpiling and foods for hot meal servings,” and “Limit the food list to stockpile-use foods that have a long shelf life.”

#### 3.1.4. Reasons for nonuse

Figure 7 shows the reasons selected by 147 individuals who indicated that they had not used (entered information into) the Simulator. The most common reason for not using the Simulator was “I do not know the information to be entered (number of estimated victims and types and amounts of stockpiled foods).” Some answered that the reason for not using the Simulator was “We don't make considerations for nutrition in the selection of foods for stockpiling”.

#### 3.1.5. Information sharing

Further, 115 participants (51.3%) stated that they had supplied information about the Simulator to the department responsible for stockpiles (disaster management department managers) and that they had been supplied with the information from the department that manages nutrition (public health department managers).

### 3.2. Group interviews

#### 3.2.1. Awareness and use of the Simulator

All 15 participants were aware of the existence of the Simulator, and Prefecture X had also accessed it. Many people in Prefecture Y had also tried the Simulator for the group interview. In Prefecture Z, one participant (city-run public health center) used it in the Japan Public Health Association workshop on November 9, 2020, whereas other (town government) tried it because of the group interview, and other (city government) saw it when the Disaster Prevention Department input the figures.

#### 3.2.2. Problems with the Simulator

The statements made regarding current problems with the Simulator and barriers to using it were categorized and shown on the basis of similarity. Examples of the statements are shown below.

Public health dietitians would not use the Simulator even if they received it.

• *Food stockpiles are purchased not by dietitians but by other staff, so it is difficult for dietitians to make proposals saying, “please buy such-and-such based on our calculations using a Simulator that we have received.”* (Y Prefecture-run Health Center)

The MHLW notice does not reach the departments responsible for stockpiling.

• *If the notice is received by the Health Department of the Prefectural Office, the only places they can contact are public health centers. Public health centers can only make transmissions through healthcare channels (e.g., municipal community healthcare centers)*. (X Prefectural Government, b)

• *If I do not directly take it to the department that handles supplies, it will only reach the emergency management supervisor in my department (healthcare and welfare). From then on, it is left to their discretion, so I think it is probably not getting as far as the emergency management departments or the departments that prepare prefectural stockpiles*. (X Prefectural Government, b)

• *If the ministries and agencies do not communicate among themselves when dealing with supplies, it probably will not be passed down any further. Rather, it would go smoothly if it were to be taken from the Cabinet Office's Disaster Management Team to the prefectural emergency management departments; thereafter, they could ask the nutrition department if they do not know how to use it*. (X Prefectural Government, a)

• *It would be better if both the nutrition department and disaster management were notified, and if the notice given to those working in disaster prevention included an additional statement asking them to consult public health dietitians when, for example, an explanation of the content is required*. (Z Prefectural Government)

Vertically compartmentalized administrative structure prevents its use.

• *If the department in charge of emergency management decides, “This has nothing to do with us, as it is the responsibility of health and welfare,” it ends there. There will be no chance for the stockpile managers and the nutrition departments to talk about it*. (X Prefectural Government, b)

• *This is the downside of a vertical administrative structure, and I think it makes things difficult*. (X Prefectural Government, a)

Content of the food-product list (Tables 2–3) is poor.

• *It is difficult (to use the Simulator) because the food-product list includes many foods that cannot be used for stockpiling*. (X City-run Public Health Center)

• *The food-product list includes dried foods; however, this cannot be used without being cooked even when stocked. If there is no information about whether a product is suitable for large stockpiles like those of a city, perhaps, there would be no affirmative decision*. (X Prefecture-run Public Health Center)

• *I would like food products that are actually used in stockpiles to be listed*. (Y Prefectural Government)

Required amounts are too large and most display “×”.

• *It makes me feel that we will definitely not have enough*. (X City Government)

• *Even when I try inputting various food products, in the end, the column of evaluation (in Figure 4) shows “×” (insufficient) for energy and all nutrients, so I think it is difficult to use this for local governments' actual stockpiling plans*. (Y Prefectural Government)

• *There are so many types of nutrients that it sets a high bar to clear*. (Y Prefectural Government)

• *Food reserves are used for 3–5 days following the outbreak of a disaster, so, to what extent should the content be considered? It is difficult to use if the bar is too high*. (Y Prefectural Government)

• *The number of columns (in Figure 4) is huge; it would be good if something like a chart or a radar chart could be provided, so that people can visualize what is lacking. If there is something that does not appeal to them, they will probably reject it just by looking at the numbers*. (Z Prefectural Government)

• *The excess/deficiency rate (in Figure 4) gives incredibly low numbers, so I repeatedly checked whether it was correct. There was no way of understanding how these values should be interpreted*. (Z City Government)

#### 3.2.3. Ideas for improvement

• *When a “×” is shown, it could be more easily conveyed to the stockpile manager by showing what and how much should be purchased*. (Y City Government)

• *Clerical workers should also be able to easily use it, and it would be useful if it showed what is lacking at a given time and what should be added when making purchases*. (Y Prefecture-run Public Health Center)

• *The food-product list should be cleaned up by limiting it to stockpile foods, using food products that are easier to understand, such as saying that protein requirements could be matched by adding canned fish as a side dish*. (Y Prefecture-run Public Health Center)

• *Everything comes up as “×”, so I would like a message that would show the priorities and essential items*. (Y Prefectural Government)

• *It would be easier to use it if it was made into a tool that could make adjustments and corrections based on the characteristics and approaches of the municipality based on a standard model for food products that are generally used for stockpiling*. (Y Prefectural Government)

• *Instead, it should show how many people can be provided with the recommended amounts using the current stock levels*. (Z City Government)

## 4. Discussion

### 4.1. Issues with stockpile provision based on feedback on the Simulator

Although the questionnaire respondents and group interview participants were aware of the existence of the Simulator, few had actually used it. The questionnaire showed that the most common reason for not using the Simulator was “I do not know the information to be entered (number of estimated victims and types and amounts of stockpiled foods)” (Figure 7). They were not aware of the types and amounts of the stockpiled foods at the local government where they worked, even though food assistance during disasters is a task included in the work of public health dietitians as specified in the “Basic Guidelines for Regional Health Promotion and the Improvement of Nutrition by Public Health Dietitians”, established by the MHLW in 2013 [26]. In a nationwide survey of municipal nutrition service staff, who are at the frontline of supporting victims, 7.3% of them responded with “I do not know” when asked whether there are specific items of water and food to be stockpiled by the municipal government and whether the amount to be stockpiled is indicated in the local disaster management plan [27]. The local disaster management plan must be prepared by all prefectures and municipalities based on the Disaster Countermeasures Basic Act [28]. These plans are produced based on the national Basic Plan for Disaster Management that is issued by the national government in accordance with the Disaster Countermeasures Basic Act. However, food stockpiles are not included among the eight key points to be emphasized in the local disaster prevention plans that are stipulated in the Basic Plan for Disaster Management, and 49.1% of municipalities made no specific comment concerning stockpiled food [27]. More than 90% of public health dietitians in public health departments are not involved in forming the local disaster management plans, which are handled by disaster management departments [29].

The responses to the questionnaire showed that one reason for not using the Simulator was “We don't make considerations for nutrition in the selection of foods for stockpiling” (Figure 7). The second most common reason for not intending to use the Simulator in the future, despite having used it before, was formulated as “Nutritionists and other professionals cannot make suggestions regarding the purchase of food stockpiles” (Figure 5), which suggests that the expertise of public health dietitians is not utilized in the selection and purchase of stockpiled food. Moreover, the group interviews found that cross-department coordination was difficult, considering that a respondent emphasized that “food stockpiles are purchased not by dietitians but by other staff, so the situation is such that it is difficult for dietitians to make proposals saying, ‘please buy such-and-such based on our calculations using a Simulator that we have received’.” The downside of Japan's vertical administration structure is striking when it comes to disasters. Under the Disaster Relief Act, in the cities, towns, and villages that are at the forefront of disaster support, there is a division of jurisdiction, with the crisis management department caring for victim relief, the livelihood and welfare department caring for evacuation shelter management, the housing department caring for temporary-housing management, and the accounting department caring for natural disaster relief fund management. Hence, disaster-affected residents cannot receive all-in-one support, and the process takes time, both of which are seen as problems [30]. The provision of meals at evacuation shelters is required from the first day of the disaster, and although it depends on the type and scale of disaster, there are many cases in which meal provision uses food stockpiles for three–five days following the disaster, as seen in the statements made in the group interviews.

Considering that the aging rate, i.e., the proportion of the population aged 65 years or above, in Japan was 28.8% in 2020, when disasters occur, food assistance for old people will constitute a large part of the provided amount [31]. Indeed, most of the fatalities related to the past disasters were old people, and nutritional deficiencies caused by unsuitable provision of meals often lead to frailty and pneumonia [32]. This has serious consequences for the lives and health of old people. Proteins are the most important nutrients for older people [33]. However, current local government food stockpiles are skewed toward carbohydrates, and only 16.4% of municipalities have side dishes providing protein in their food stockpiles [34]. Even the Simulator's food list states that “meals based on staple foods lack nutritional balance, so it is important to ensure nutrition by also having canned foods and other side dishes in stock” (Table 3), and the form allows food products to be selected for both staple foods and main/side dishes (Figure 3). However, this list of main/side dish foods was not well received. One of the reasons given for not planning to use the Simulator in the future was that “the food list contains many foods that are perishable or that require cooking, and are not suitable for stockpiling” (Figure 5), and many participants in the group interviews remarked that the content of the food list was poor.

### 4.2. Areas of improvement for the Simulator

Answers regarding the food list were also chosen as ideas for improving the Simulator, such as “Provide separate lists for foods for stockpiling and foods for hot meal services,” and “Limit the food list to stockpile-use foods that have a long shelf life” (Figure 6).

In the group interviews, there were comments such as “this cannot be used without being cooked even when stocked” and “I would like food products that are actually used in stockpiles to be listed”. Therefore, in terms of improvements to the Simulator, which was one of the objectives of our study, one suggestion would be a revision of the list of foods.

There were also comments that the number of digits shown in the amount of energy and nutrients required and supplied by the current stockpile (Figure 4) was too large to determine what they meant. Both the questionnaire and group interviews indicated that if “×” is shown next to everything when evaluating current reserves, it erodes the motivation to make improvements, which leads people to stop using the Simulator itself. As the values of Dietary Reference Intakes (DRIs) for Japanese 2015 used in calculating the rates of excess/deficiency cannot be changed, the number of nutrients shown could be reduced, thereby lowering barriers to entry. The nutrients highlighted by the MHLW in “Nutritional Reference Values to be used as Near-term Targets for the Planning and Assessment of Meal Provision in Evacuation Shelters for Three Months Following a Disaster,” which were sent out to disaster-hit prefectures one month after the Great East Japan Earthquake, were calories; protein; and vitamins B_1_, B_2_, and C. These nutrients are particularly important at the time of a disaster. Among these, vitamins B_1_ and B_2_ are required to metabolize carbohydrates, and while there is a great need for them because of the carbohydrate-based diets at evacuation shelters, such as rice balls and pastries, the fact that they are contained in the main dishes means that they are often in short supply [35]. However, it has been confirmed in prior studies that it is possible to fulfill the need for these five nutrients just by processed foods that require no preparation and that can be stored at room temperature [36].

Nevertheless, even if the nutritional display is limited to just these five nutrients, nutrients other than calories cannot be supplied only by stockpiles of staple foods [37]. Therefore, these “×” would still be displayed. Accordingly, the Simulator could be used to show the combination of foods that are suitable for stockpiling, or can be easily visualized as parts of meals, and meet the daily requirements for one person (i.e., an excess/deficiency rate of 100%), and then exclude foods that cannot be stockpiled with respect to the budget and storage space available to the municipality. This would allow people to understand which nutrients' percentages would decrease if certain foods were removed. The current Simulator is a tool for assessing current stockpiles and not a meal-planning tool for selecting foods for stockpiling. It was also indicated that if the nutritional values rather than amounts of food were displayed, the administrative staff in charge of disaster management, who have no expertise in nutrition and food, would not be able to understand it (Figure 5). By displaying ideal stockpiling foods in specific combinations, the Simulator would be easier to use for towns and villages that do not have public health dietitians. The NutVal Excel sheet, first developed by the United Nations High Commissioner for Refugees in the 1990s, is software that enables the calculation of nutritional values of relief foods. The Ration Calculator function in Version 4 shows three examples, namely, wheat-, maize-, and rice-based rations, and the food products contained within them can be changed [38]. Improvements should be made with reference to such tools from international organizations.

If the Simulator's functionality as an assessment tool is to be retained, one idea for improvement suggested in the group interviews was that the types and amounts of current food reserves could be shown in terms of the number of people that could be covered according to the DRIs. In this case, while it is expected that this may not be enough for the number of estimated victims stated in local disaster prevention plans, the separate displaying of each nutrient may make it clear that other nutrients cannot be supplied without side dishes, even though the caloric values may cover a relatively large number of people. In brief, it has been suggested that the number of people covered rather than a percentage value which is difficult even for dieticians, may be easier to interpret.

Based on the survey results of local government staff all over Japan regarding the Simulator and group interviews with public health dietitians in disaster-affected areas, it was found that a vertical administrative structure made it difficult to use the expertise of public health dietitians to establish food stockpiles that take nutrition into consideration. Moreover, it was shown that with current food reserves, which are carbohydrate-based and low in quantity, judgments made using the Simulator are always negative, which do not lead to specific improvements. In order for the administrative staff in charge of disaster management to understand the nutritional importance of stockpiling main/side dishes, it was suggested that specific combinations of foods that meet the required amounts should be shown and that visualization using food products and the number of people covered by stockpiled foods may be easier to understand than pure nutritional values. Accordingly, we plan to improve the Simulator as the next step and ascertain whether appropriate improvements have been made by testing the improved Simulator with the group interview participants and other local government disaster management staff.

## 5. Conclusions

More than 70% of respondents were aware of the existence of the Simulator while only 36.6% of them used the Simulator. In order for the administrative staff in charge of disaster management to understand the nutritional importance of stockpiling main/side dishes, it was suggested that specific combinations of foods that meet the required amounts should be shown and that visualization using food products and the number of people covered by stockpiled foods may be easier to understand than pure nutritional values.
